# TUBectomy with delayed oophorectomy as an alternative to risk-reducing salpingo-oophorectomy in high-risk women to assess the safety of prevention: the TUBA-WISP II study protocol

**DOI:** 10.1136/ijgc-2023-004377

**Published:** 2023-04-12

**Authors:** Miranda P Steenbeek, Majke H D van Bommel, Joanna intHout, Christine B Peterson, Michiel Simons, Kit C B Roes, Marleen Kets, Barbara M Norquist, Elizabeth M Swisher, Rosella P M G Hermens, the TUBA-WISP II consortium, Karen H Lu, Joanne A de Hullu

**Affiliations:** 1 Obstetrics & Gynaecology, Radboudumc, Nijmegen, The Netherlands; 2 Department for Health Evidence, Radboudumc, Nijmegen, The Netherlands; 3 Department of Biostatistics, The University of Texas MD Anderson Cancer Center, Houston, Texas, USA; 4 Patholoy, Radboudumc, Nijmegen, The Netherlands; 5 Human Genetics, Radboudumc, Nijmegen, The Netherlands; 6 Obstetrics & Gynecology, University of Washington, Seattle, Washington, USA; 7 Scientific Institute for Quality of Healthcare, Radboudumc, Nijmegen, The Netherlands; 8 Radboudumc, Nijmegen, The Netherlands; 9 Gynecologic Oncology & Reproductive Medicine, The University of Texas MD Anderson Cancer Center, Houston, Texas, USA

**Keywords:** Ovarian Cancer, BRCA1 Protein, BRCA2 Protein, Gynecologic Surgical Procedures, Carcinoma

## Abstract

**Background:**

Risk-reducing salpingectomy with delayed oophorectomy has gained interest for individuals at high risk for tubo-ovarian cancer as there is compelling evidence that especially high-grade serous carcinoma originates in the fallopian tubes. Two studies have demonstrated a positive effect of salpingectomy on menopause-related quality of life and sexual health compared with standard risk-reducing salpingo-oophorectomy.

**Primary Objective:**

To investigate whether salpingectomy with delayed oophorectomy is non-inferior to the current standard salpingo-oophorectomy for the prevention of tubo-ovarian cancer among individuals at high inherited risk.

**Study Hypothesis:**

We hypothesize that postponement of oophorectomy after salpingectomy, to the age of 40–45 (*BRCA1*) or 45–50 (*BRCA2*) years, compared with the current standard salpingo-oophorectomy at age 35–40 (*BRCA1*) or 40–45 (*BRCA2*) years, is non-inferior in regard to tubo-ovarian cancer risk.

**Trial Design:**

In this international prospective preference trial, participants will choose between the novel salpingectomy with delayed oophorectomy and the current standard salpingo-oophorectomy. Salpingectomy can be performed after the completion of childbearing and between the age of 25 and 40 (*BRCA1*), 25 and 45 (*BRCA2*), or 25 and 50 (*BRIP1, RAD51C,* and *RAD51D* pathogenic variant carriers) years. Subsequent oophorectomy is recommended at a maximum delay of 5 years beyond the upper limit of the current guideline age for salpingo-oophorectomy. The current National Comprehensive Cancer Network (NCCN) guideline age, which is also the recommended age for salpingo-oophorectomy within the study, is 35–40 years for *BRCA1*, 40–45 years for *BRCA2,* and 45–50 years for *BRIP1*, *RAD51C*, and *RAD51D* pathogenic variant carriers.

**Major Inclusion/Exclusion Criteria:**

Premenopausal individuals with a documented class IV or V germline pathogenic variant in the *BRCA1, BRCA2, BRIP1, RAD51C,* or *RAD51D* gene who have completed childbearing are eligible for participation. Participants may have a personal history of a non-ovarian malignancy.

**Primary Endpoint:**

The primary outcome is the cumulative tubo-ovarian cancer incidence at the target age: 46 years for *BRCA1* and 51 years for *BRCA2* pathogenic variant carriers.

**Sample size:**

The sample size to ensure sufficient power to test non-inferiority of salpingectomy with delayed oophorectomy compared with salpingo-oophorectomy requires 1500 *BRCA1* and 1500 *BRCA2* pathogenic variant carriers.

**Estimated Dates for Completing Accrual and Presenting Results:**

Participant recruitment is expected to be completed at the end of 2026 (total recruitment period of 5 years). The primary outcome is expected to be available in 2036 (minimal follow-up period of 10 years).

**Trial Registration Number:**

NCT04294927.

## Introduction

In recent years, risk-reducing salpingectomy with delayed oophorectomy has gained interest as a novel strategy to prevent tubo-ovarian cancer among individuals at high inherited risk.^1^ Tubo-ovarian cancer is a collective term for carcinomas of the ovaries, the fallopian tubes, and the peritoneum. Several gene germline pathogenic variants are known to cause an increased risk of tubo-ovarian cancer, such as pathogenic variants in the *BRCA1, BRCA2, RAD51C, RAD51D,* and *BRIP1* genes.^2^ Currently, individuals with these pathogenic variants are advised to undergo simultaneous removal of both fallopian tubes and ovaries (risk-reducing salpingo-oophorectomy) at the age of 35–40 (*BRCA1*), 40–45 (*BRCA2*), or 45–50 (*BRIP1, RAD51C, RAD51D*) years.^3^ Salpingo-oophorectomy decreases the risk of tubo-ovarian cancer, yet also results in a surgical menopause in those women who are premenopausal.^4^ The acute loss of estrogen exposure through salpingo-oophorectomy can induce short-term symptoms such as vasomotor complaints, sleep disturbances, sexual problems, and potential long-term adverse effects including osteoporosis, cardiovascular disease, and impaired neurocognitive functioning.^4^ Those side effects can be frequently alleviated by hormone replacement therapy, which is generally recommended to use after salpingo-oophorectomy.^5^

There is compelling evidence that high-grade serous carcinoma, the most frequent histological subtype of tubo-ovarian cancer, originates in the fallopian tubes.^6–8^ Therefore, the focus of preventing tubo-ovarian cancer has shifted towards the fallopian tubes instead of the ovaries.^1^ Potentially, salpingectomy with delayed oophorectomy could prevent tubo-ovarian cancer on the one hand and delay surgical and premature menopause on the other.^9^ Recently, two prospective studies, TUBA and WISP, demonstrated that participants after salpingectomy had a better menopause-related quality of life and sexual health when compared with patients after salpingo-oophorectomy.^10 11^ No cases of tubo-ovarian cancer after salpingectomy have been detected in either study to date, but neither was powered to demonstrate the efficacy of salpingectomy for the prevention of tubo-ovarian cancer. In 2016, Harmsen et al estimated tubo-ovarian cancer risk after salpingectomy by using a simulation model, to guide participants choosing between both preventive strategies in the earlier mentioned studies.^9^ Thus far, no previous studies have addressed the efficacy and safety of salpingectomy with delayed oophorectomy because of the large number of participants and long follow-up needed for these objectives.

As the safety is not yet proven, salpingectomy with delayed oophorectomy for primary prevention should only be offered within the setting of a clinical trial. Previous feasibility studies and experiences in both TUBA and WISP have demonstrated that high-risk individuals are not willing to be randomized.^12 13^ Therefore, the investigators from these major studies have collaborated to design an international prospective preference study to evaluate the novel treatment as an alternative to the standard treatment in participants with a *BRCA1/2* pathogenic variant with respect to tubo-ovarian cancer incidence. We hypothesize that postponement of oophorectomy after salpingectomy, to the age of 40–45 (*BRCA1*) or 45–50 (*BRCA2*) years, compared with the current standard salpingo-oophorectomy at age 35–40 (*BRCA1*) or 40–45 (*BRCA2*) years, is non-inferior in regard to tubo-ovarian cancer risk.

## Methods

### Trial Design

**Figure 1:**
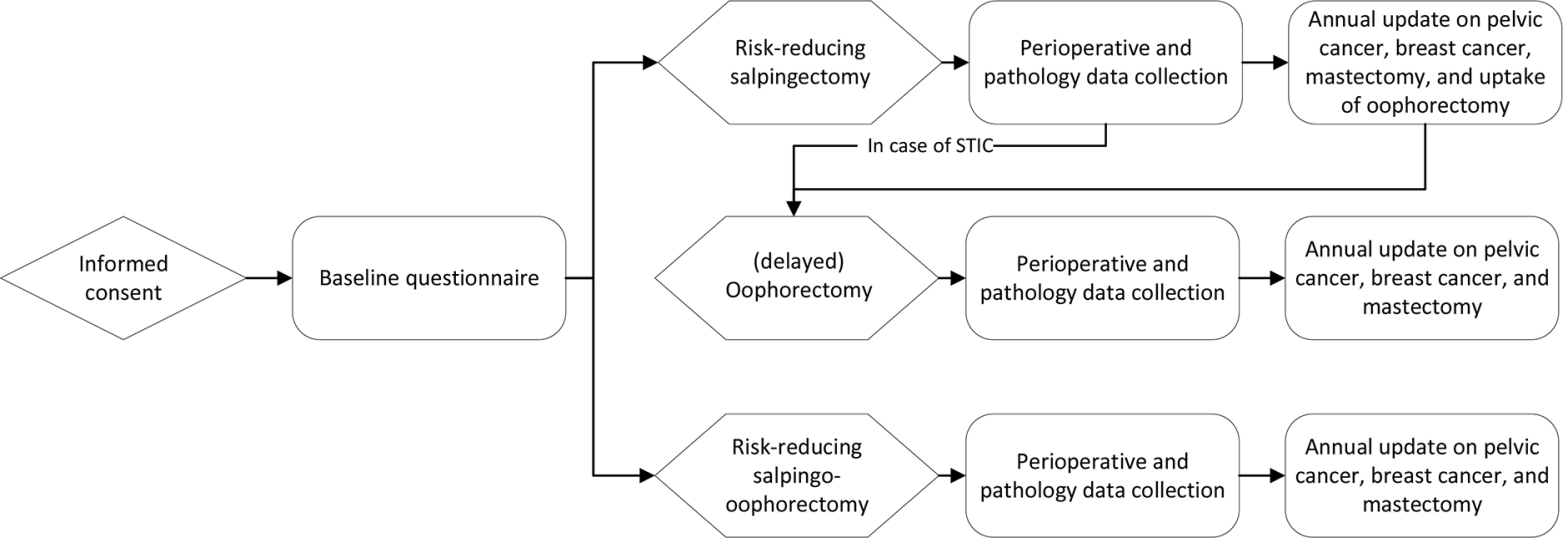
Study design. STIC, serous tubal intraepithelial carcinoma.

The TUBA-WISP II study has a prospective, preference design with two treatment arms: risk-reducing salpingo-oophorectomy and risk-reducing salpingectomy with delayed oophorectomy. Participants choose their preferred treatment. This study will be multisite and international, and about 35 centers worldwide have expressed their intention to participate. Funding for study start-up is available in The Netherlands (Dutch Cancer Society) and in the USA (Any Mountain). Figure 1 provides an overview of the study design.

### Participants

Inclusion and exclusion criteria are shown in Table 1. Participants of the TUBA study (n=577) will be included in the study population for TUBA-WISP II. WISP participants will not be included as that study used slightly different inclusion criteria and participants were not counseled for oophorectomy beyond the current guideline ages.

**Table 1:** Inclusion and exclusion criteria

Inclusion criteria	Exclusion criteria
Premenopausal status	Wish for second-stage oophorectomy within 2 years after salpingectomy (if clear at enrollment)
Documented class IV or V germline pathogenic variant in the *BRCA1*, *BRCA2*, *BRIP1*, *RAD51C*, or *RAD51D* gene	Prior bilateral salpingectomy
Age 25–40 years for *BRCA1*, 25–45 years for *BRCA2,* and 25–50 years for *BRIP1*, *RAD51C*, and *RAD51D* pathogenic variant carriers	Personal history of ovarian, fallopian tube, or peritoneal cancer
Childbearing completed	Current clinical signs, diagnosis, or treatment for malignant disease
Presence of at least one fallopian tube	
Participants may have a personal history of a non-ovarian malignancy	

### Primary Endpoints

**Figure 2:**
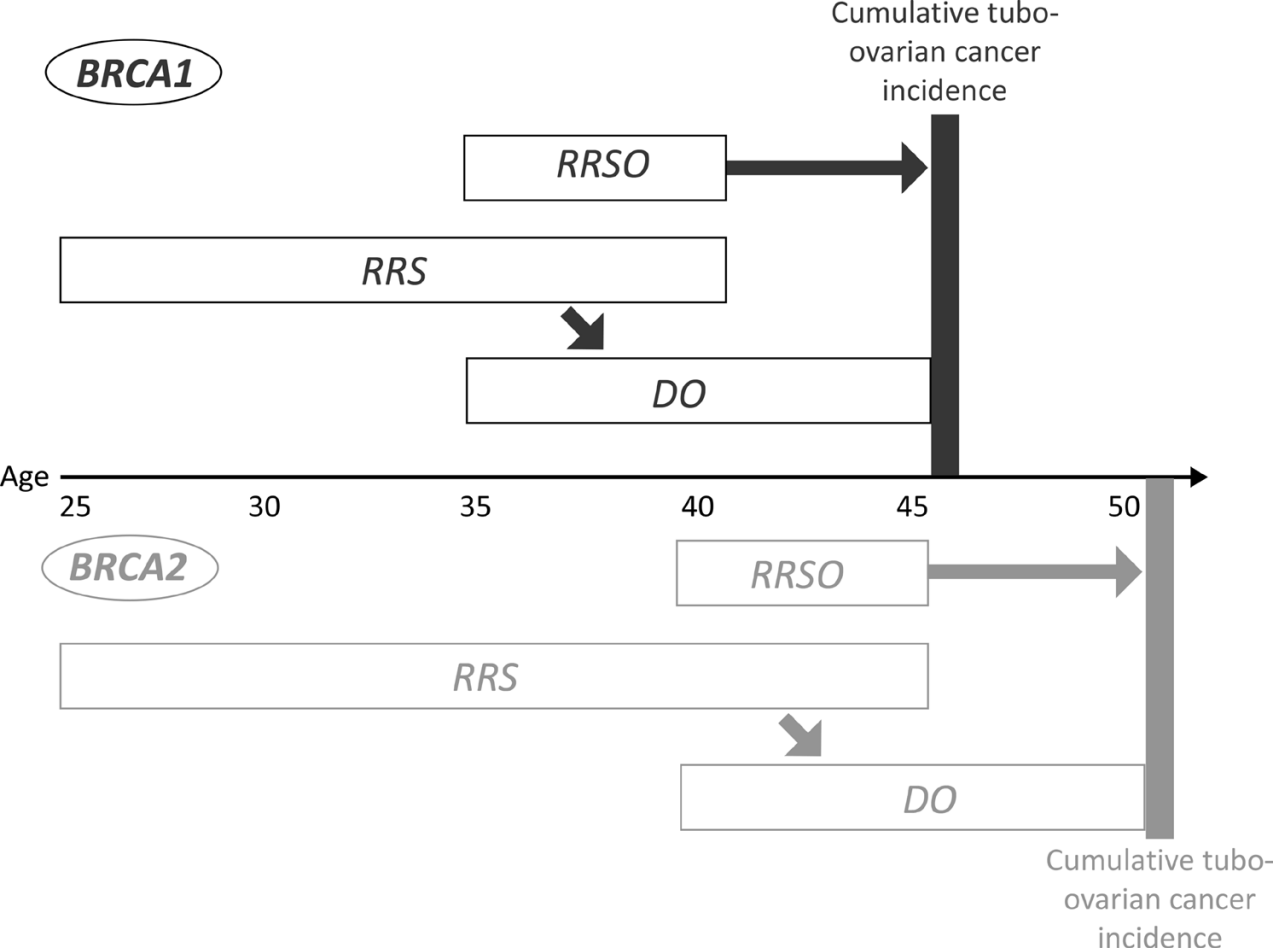
Primary outcome of cumulative tubo-ovarian cancer incidence. DO, delayed oophorectomy; RRS, risk-reducing salpingectomy; RRSO, risk-reducing salpingo-oophorectomy.

The primary outcome is the cumulative tubo-ovarian cancer incidence at target age: 46 years for *BRCA1* and 51 years for *BRCA2* pathogenic variant carriers, as visualized in Figure 2. As the risk for tubo-ovarian cancer increases with age, use of a certain period of follow-up, for example, 10 years since inclusion, would lead to an assumed advantage for the novel treatment arm. Additional considerations regarding the rationale for the primary outcome are described in the Online Supplemental Material. We assume that after salpingo-oophorectomy and delayed oophorectomy, with normal pathology results, the differences in tubo-ovarian cancer risk across the treatment groups are similar. Individuals with a pathogenic variant in one of the moderate risk genes (*RAD51C, RAD51D, BRIP1*) are eligible for participation, but will not be included in the primary analysis because of their low prevalence and lower tubo-ovarian cancer risk.

10.1136/ijgc-2023-004377.supp1 Supplementary data

The secondary outcomes are the incidence of (pre)malignancies in fallopian tubes and/or ovaries, the incidence of pelvic cancer, the incidence of breast cancer, peri-operative morbidity and mortality, and uptake and results of prophylactic breast surgery and oophorectomy.

### Sample Size

A total of 1500 *BRCA1* and 1500 *BRCA2* pathogenic variant carriers (in total 3000) are needed to ensure sufficient power to test non-inferiority of salpingectomy with delayed oophorectomy compared with salpingo-oophorectomy regarding tubo-ovarian cancer incidence. Individuals with a pathogenic variant in a moderate risk gene (*RAD51C, RAD51D, BRIP1*) are eligible for participation, but do not contribute to the sample size as they are excluded from the primary analysis.

Sample size considerations are based on: (1) the primary endpoint of cumulative tubo-ovarian cancer incidence; (2) the target age is 46 years for *BRCA1* and 51 years for *BRCA2* pathogenic variant carriers; (3) at target age, the cumulative tubo-ovarian cancer incidence for participants undergoing salpingo-oophorectomy is assumed to be 1.0% for *BRCA1* and 0.5% for *BRCA2* pathogenic variant carriers; (4) at target age, cumulative tubo-ovarian cancer incidence is equal for participants undergoing salpingectomy with delayed oophorectomy and participants undergoing salpingo-oophorectomy; (5) the ratio between salpingectomy with delayed oophorectomy and salpingo-oophorectomy is expected to be between 1:1 and 2:1, based on recruitment ratios in the TUBA and WISP studies; (6) the ratio between *BRCA1* and *BRCA2* pathogenic variants is expected to vary between 1:1 and 2:1, based on recruitment ratios in the TUBA and WISP studies; (7) an expected drop-out rate of 10% (the current drop-out rates in TUBA and WISP are approximately 3%; a higher rate is anticipated due to the long study duration); and (8) non-inferiority is reached when the upper limit of the one-sided 97.5% CI for the difference in cumulative incidence between salpingectomy with delayed oophorectomy and salpingo-oophorectomy is ≤2.0% for *BRCA1* and ≤1.5% for *BRCA2* pathogenic variant carriers.

For the sample size calculations, we considered the cumulative tubo-ovarian cancer incidence at target ages as a binomial outcome, given the fact that the proportional hazards assumption may not hold for the comparison between the salpingectomy with delayed oophorectomy versus the salpingo-oophorectomy and in view of the many variations in timing of salpingectomy, oophorectomy, and salpingo-oophorectomy. Table 2 shows a study with 3000 participants, that is, 1500 *BRCA1* and 1500 *BRCA2* pathogenic variant carriers is sufficient to have at least 86% power in all scenarios.

**Table 2:** Possible scenarios of treatment ratios with corresponding number of participants and corresponding power

	Ratio salpingectomy with delayed oophorectomy:salpingo-oophorectomy	Salpingectomy with delayed oophorectomy (N)	Salpingo-oophorectomy (N)	Total (N)	Total with 10% dropout added (N)	Power (%)
*BRCA1*	1:1	675	675	1350	1500	89
	2:1	900	450	1350	1500	92
*BRCA2*	1:1	675	675	1350	1500	86
	2:1	900	450	1350	1500	91

### Statistical Methods

The primary outcome of cumulative tubo-ovarian cancer incidence at the target age will be analyzed per *BRCA*-type using Kaplan-Meier analysis with stabilized inverse probability of treatment weighting (IPW). The stabilized IPW weights, used to adjust for possible imbalances, will be defined based on a logistic regression model, using as the dependent variable the actual treatment, and as independent variables possible confounders, that is, at least: age at inclusion, history of breast cancer, family history of tubo-ovarian or breast cancer, and region (United States, European Union, Australia). Each observation will be weighted with its own stabilized IPW weight given the observed values of the confounders.

Subsequently, we will estimate the difference in cumulative tubo-ovarian cancer incidence between the salpingectomy with delayed oophorectomy group minus the salpingo-oophorectomy group for the *BRCA1* and the *BRCA2* pathogenic variant carriers, and determine one-sided 97.5% CIs (or equivalently 95% two-sided CIs) for these differences. In case of dropout (loss-to-follow up or death due to causes other than tubo-ovarian cancer) in the evaluation period defined above, this timepoint will be treated as “censored” in the analysis. For the non-inferiority assessment, we assume that the cumulative tubo-ovarian cancer risk in *BRCA1* at age 46 years is 1% and in *BRCA2* pathogenic variant carriers at age 51 years is 0.5%, and are equal in both treatment arms. In order to define the non-inferiority margins, a small added risk of maximum 1.5–2% is considered allowable in view of the gains in quality of life under salpingectomy with delayed oophorectomy and the residual error in the estimated cumulative risks. Consequently, the upper limit of the one-sided 97.5% CI of the risk difference will be compared with the non-inferiority margin of 2% for *BRCA1* and 1.5% for the *BRCA2* pathogenic variant carriers. If the upper limit is below this margin, we conclude that the salpingectomy with delayed oophorectomy method is non-inferior to the salpingo-oophorectomy method with respect to tubo-ovarian cancer incidence. It is noted that to successfully demonstrate non-inferiority under the presented assumptions and across the sample size scenarios below, the estimated difference in incidence for *BRCA1* pathogenic variant carriers always needs to be smaller than 0.9%, and for *BRCA2* pathogenic variant carriers smaller than 0.07% (based on binomial approximation, with CI just borderline below 2% and 1.5%, respectively). As the aim is to demonstrate non-inferiority for each type of pathogenic variant separately, no correction for multiple testing is deemed necessary.

## Discussion

In this study protocol, we describe an international prospective non-inferiority study with a preference design, investigating the oncological safety of risk-reducing salpingectomy with delayed oophorectomy compared with the standard risk-reducing salpingo-oophorectomy in individuals at high inherited risk for tubo-ovarian cancer.

The TUBA and WISP studies clearly demonstrated the positive effects of delaying menopause on menopause-related quality of life and sexual health. However, the efficacy for tubo-ovarian cancer prevention has not yet been elucidated. Oncological safety is the most important prerequisite to allow implementation of salpingectomy with delayed oophorectomy in clinical practice. The study of LeBlanc et al on the radical fimbriectomy has shown promising results, although the sample size in that study is far too small to draw conclusions on safety.^14^

At present there are four major reasons to discourage salpingectomy with delayed oophorectomy outside the setting of a clinical trial. (1) Uniform and expert counseling to clearly state the current knowledge and uncertainty in tubo-ovarian cancer risk reduction, so participants can make an informed choice that suits their personal situation. (2) Strict age limits as, theoretically, salpingectomy is most effective when performed as early as possible. The average age of diagnosis of high-grade serous carcinoma is 51–53 years for *BRCA1* and 55–60 years for *BRCA2* pathogenic variant carriers^15^ and evolutionary analysis demonstrated a 7-year interval between serous tubal intraepithelial carcinoma and invasive high-grade serous carcinoma.^7 8^ Therefore, we hypothesize that salpingectomy is most effective when performed at a maximum age of 40 years for *BRCA1* and 45 years for *BRCA2* pathogenic variant carriers. (3) Standardized and thorough pathology by using the SEE-FIM (sectioning and extensively examining the fimbriated end) protocol and performed by an experienced pathologist. When a serous tubal intraepithelial carcinoma is found it is important to perform oophorectomy at short notice, as the serous tubal intraepithelial carcinoma could be accompanied by an invasive carcinoma in the ovary. This was observed in the TUBA study in a 42-year-old *BRCA2* pathogenic variant carrier with a serous tubal intraepithelial carcinoma lesion in the fallopian tube and an invasive high-grade serous carcinoma at subsequent oophorectomy and staging surgery (Stage IA).^10^ Thus, missing a serous tubal intraepithelial carcinoma could expose a participant to high-stage tubo-ovarian cancer. (4) Close monitoring of the tubo-ovarian cancer incidence with a safety rule, as all participants are prospectively registered; hereby there is an early warning possible in case of an unexpected high incidence of tubo-ovarian cancer after salpingectomy. In that case, we know exactly which participants are at risk so they can be informed of the new situation. In future, it would be ideal if second-stage oophorectomy does not have to be performed at all. However, some ovarian inclusion cysts are lined by epithelial cells resembling fallopian tube epithelium. Previously, we observed malignant transformation of such inclusion cysts. Therefore, at this moment, monitoring performance of oophorectomy is essential.

There are two other studies internationally evaluating risk-reducing salpingectomy in high-risk individuals. The first study, PROTECTOR, has been recruiting since 2018 in the United Kingdom. This is a three-armed study comparing salpingectomy with delayed oophorectomy, salpingo-oophorectomy, and no surgery to investigate sexual health (ISRCTN25173360). The second study, SOROCk, has been recruiting since 2020 in the United States and is comparing salpingectomy with delayed oophorectomy to salpingo-oophorectomy to investigate the time to development of incident high-grade serous carcinoma (NCT04251052). In the latter study, only individuals ages at least 35 yearswith a *BRCA1* pathogenic variant who decline salpingo-oophorectomy after counseling are included for the salpingectomy arm and can choose to undergo oophorectomy. Even though the latter study looks similar to the TUBA-WISP II study, the studies investigate different strategies. The strategy in SOROCk is aimed at individuals declining salpingo-oophorectomy, while the strategy in TUBA-WISP II is aimed at participants choosing between the two surgeries, and at a younger age. The maximum age at inclusion is 50 years in SOROCk and 40 years for *BRCA1* pathogenic variant carriers in TUBA-WISP II. Given the potential for high-grade neoplastic lesions in the fallopian tube to disseminate quickly to the ovary, even prior to invasion, salpingectomy is potentially more effective when performed at a younger age. Moreover, the age limit for oophorectomy is not defined in SOROCk: oophorectomy is recommended at the standard age of 40 years, although not mandated since women can enroll beyond that age. In contrast, in TUBA-WISP II, oophorectomy can be maximally delay with 5 years after the current upper limit of the recommended age in TUBA-WISP II. Thus, the strategies of prevention are different on a few crucial points, making both studies distinctly different.

The novel strategy of salpingectomy with delayed oophorectomy has been shown to increase menopause-related quality of life and sexual health compared with salpingo-oophorectomy up until 1 year after surgery while offering a potentially protective effect of salpingectomy. Long-term follow-up on quality of life, sexual health, cost-effectiveness, and cardiovascular disease will be gathered within the TUBA and WISP studies.

To conclude, the TUBA-WISP II study compares a novel strategy of risk-reducing salpingectomy with delayed oophorectomy to risk-reducing salpingo-oophorectomy in order to investigate the oncological safety with respect to tubo-ovarian cancer incidence. In the current collaborative TUBA-WISP II study, we hypothesize that salpingectomy with delayed oophorectomy is non-inferior to salpingo-oophorectomy regarding cumulative tubo-ovarian cancer incidence until the age of 46 years for *BRCA1* and 51 years for *BRCA2* pathogenic variant carriers.

## Data Availability

There are no data in this work.
