# Supplementary material for: TUBectomy with delayed oophorectomy as an alternative to risk-reducing salpingo-oophorectomy in high-risk women to assess the safety of prevention: the TUBA-WISP II study protocol
Source: Int J Gynecol Cancer. 2023 Apr 12;33(6):982–7. doi: 10.1136/ijgc-2023-004377 (PMC10314019; doi:10.1136/ijgc-2023-004377)
Supplement: Supplementary data [file ijgc-2023-004377supp001.pdf]

Supplementary Methods

Participants

Eligible potential participants will be informed about the TUBA-WISP II study during consultation at the clinical genetics or at the gynecology department. Also, potential participants will be informed via hospital websites, patient federations, social media, and family members. Eligible potential participants will be counselled by a gynecologist or gynecologic oncologist and will receive a patient information form and a decision aid.[1, 2]

Participants should be premenopausal as the main advantage of the novel treatment is delaying surgical menopause. At inclusion, participants should aim to delay oophorectomy at least two years as in a smaller time period the positive effects on menopause-related outcomes are expected to be minimal and will not outweigh the potential side-effects of two surgeries within that time period. Childbearing should be completed or not desired. In case of a previous malignancy, participants should have completed treatment (including for example tamoxifen) in order to have similar inclusion criteria to the TUBA study.

Interventions

Standard treatment

Risk-reducing salpingo-oophorectomy at age 35-40 years in *BRCA1,* 40-45 years in *BRCA2,* and 45-50 years in *BRIP1, RAD51C* and *RAD51D* pathogenic variant carriers after completion of childbearing.

Novel treatment

Risk-reducing salpingectomy upon completion of childbearing, but within the age range of 25-40 for *BRCA1*, 25-45 for *BRCA2*, and 25-50 for *BRIP1, RAD51C,* and *RAD51D* pathogenic variant carriers. Delayed oophorectomy is advised at a maximum age of 45 for *BRCA1*, 50 for *BRCA2,* and 55 for *BRIP1*, *RAD51C,* or *RAD51D* pathogenic variant carriers.

Surgery (either salpingectomy, oophorectomy, or salpingo-oophorectomy)

Standard laparoscopy as per surgical protocol with obtainment of abdominal washing and extensive exploration of the abdominopelvic cavity, if necessary, performance of surgery by laparotomy is permitted if needed, but vaginal surgery is not permitted since inspection of the abdominopelvic cavity is not possible with this approach. Abdominal washing should be obtained from either present

ascites/fluid or by flushing with 20mL fluid and regain by aspiration of 10 mL of fluid. Extensive exploration of the abdominopelvic cavity includes at least an inspection of the peritoneum, omentum, diaphragm, liver, and pelvis. Tissues should be marked left/right.

Tissues have to be totally embedded for pathological examination; the fallopian tubes must be embedded in conformity with the Sectioning and Extensively Examining the FIMbriated End (SEE-FIM) protocol.[3] Tissue will be firstly assessed on regular hematoxylin & eosin staining. When the epithelium shows cytological atypia, immunohistochemistry with p53 and Ki-67 staining will be performed additionally. In case of a serous tubal intraepithelial carcinoma (STIC) in the fallopian tube of a salpingectomy specimen, oophorectomy within short notice is advised. A panel consisting of gynecologic pathologists is available for consultation in case of difficult cases.

Data collection

All data will be collected in a secure web-based electronic database including questionnaires and case report forms. At inclusion, the treating physician will complete a case report form to provide information on in- and exclusion criteria and baseline characteristics. The participants will provide their baseline characteristics by filling in a web-based electronic questionnaire with questions regarding their medical history, family history, and use of medication. Within three months after surgery, pathological and surgical outcomes will be reported by the treating physician by completing the corresponding form. Long-term follow-up will consist of annual updates including the incidence of pelvic cancer, breast cancer, and uptake and results of prophylactic breast surgery, and of oophorectomy in case of earlier salpingectomy. Three methods of follow-up are allowed, depending on the specific policy and facilities in participating countries and institutes. First, annual out-patient visit in which the results are provided in a case report form by the treating physician, transvaginal ultrasound and blood tests (i.e., CA125) can be added depending on local policy. As second method, a yearly questionnaire is sent to the participant. As third method, a nationwide pathology database is assessed yearly and used to complete a case report form. An example of this third method is used in the Netherlands, the Pathological-Anatomical National Automated Archives is used as it includes all pathological assessments of tissues in the Netherlands.[4] The third method is combined with a yearly check in the municipal personal records database to assess whether participants are still alive. In case of death, the cause of death will be determined from the central bureau of statistics.

Rationale for the Primary Endpoint

The primary endpoint is set on target age 46 (*BRCA1*) and 51 (*BRCA2*) as we observed an age difference at inclusion of 1.6 years in *BRCA1* and 2.8 years in *BRCA2* pathogenic variant carriers (due to different guideline ages for salpingo-oophorectomy) between both treatment arms in the combined data from the TUBA and WISP study. Some participants, especially *BRCA2* pathogenic variant carriers, might thus be substantially younger at salpingectomy than participants at salpingo-oophorectomy. As the risk for tubo-ovarian cancer increases with age, use of a certain period of follow-up, e.g., 10 years since inclusion, would lead to an assumed advantage for the salpingectomy group. Tubo-ovarian cancers will be counted from inclusion onwards to deal with possible imbalance in waiting times between inclusion and first surgery, e.g., in case of limited surgery sessions, participants below the guideline age might be longer on a waiting list, and as participants choosing salpingectomy are younger, waiting time might depend on the treatment strategy. To determine the target age, we assumed that after oophorectomy the risk of tubo-ovarian cancer is similar to the risk after salpingo-oophorectomy. The preferred timepoint to evaluate non-inferiority is when the contrast between both treatment arms is assumed to be the largest; shortly after the recommended age of oophorectomy. Meaning, the contrast will be maximal at the age of 45 for *BRCA1* and 50 for *BRCA2* pathogenic variant carriers. However, the salpingo-oophorectomy surgery is conducted at the latest at age 40 (45 for *BRCA2*) whereas the oophorectomy surgery might be conducted till age 45 (50 for *BRCA2*). Surgery is a moment in which early-stage ovarian cancers might be detected and the salpingo-oophorectomy patient of 45 (50 for *BRCA2*) will not undergo surgery at that age. Therefore, we added one additional year which is considered to be sufficient to also detect peritoneal carcinomatosis based on clinical symptoms (latent at age 45 for *BRCA1* or 50 for *BRCA2*) in the salpingo-oophorectomy group (Figure 2). We assume that after salpingo-oophorectomy and oophorectomy the differences in tubo-ovarian cancer risk across the treatment groups are similar.

Safety

A Data Safety Monitoring Board (DSMB) is established with four independent medical specialists who have no conflict of interest and agree with the outline of the protocol. Meetings are organized annually, and additional meetings are planned in case of the occurrence of interval carcinomas (tubo-ovarian cancers diagnosed in the interval between salpingectomy and oophorectomy) to assess whether the safety rule is met. The safety rule is implemented to flag early in case of a potential higher rate of invasive cancers as previously expected in participants undergoing the novel treatment (salpingectomy). The safety rule is based on an annual evaluation of observed tubo-ovarian cancers in the novel salpingectomy with delayed oophorectomy group only. Monitoring of the novel

treatment group only is expected to be more sensitive, since direct comparison of treatment arms would require adjustment for confounding, and incidence is expected to be very low, which is thus unfeasible early in the study. If the rule is met, study enrolment will be put on hold and a meeting of the DSMB will be convened to analyse and discuss tubo-ovarian cancer cases and safety of the study. The DSMB will provide independent advice to the principal investigators and may recommend changes in the conduct of the study or premature termination.

Ethical considerations and participating sites

The study will be conducted in accordance with the principles of the Declaration of Helsinki and the Medical Research Involving Human Subjects Act. The study protocol has gained medical-ethical approval by the Medical-Ethical Committee of Arnhem-Nijmegen (NL 50048.091.14) and Institutional Review Board of the MD Anderson Cancer center. Sites that have gained approval as well are:

- Maastricht University Medical Center, Maastricht, the Netherlands
- Catharina Hospital, Eindhoven, the Netherlands
- Elisabeth-TweeSteden Hospital, Tilburg, the Netherlands
- Erasmus MC Cancer Clinic, Rotterdam, the Netherlands
- Leiden University Medical Centre, Leiden, the Netherlands
- AmsterdamUMC, Amsterdam, the Netherlands
- Netherlands Cancer Institute/Antoni van Leeuwenhoek Hospital, Amsterdam, the Netherlands
- University Medical Centre Groningen, Groningen, the Netherlands
- UMC Utrecht Cancer Centre, Utrecht, the Netherlands
- Maxima Medical Centre, Veldhoven, the Netherlands
- Medical Centre Leeuwarden, Leeuwarden, the Netherlands
- Medical Spectrum Twente, Enschede, the Netherlands
- Isala Hospital, Zwolle, the Netherlands
- Amphia Hospital, Breda, the Netherlands
- MD Anderson, Houston, Texas, USA
- Oslo University Hospital, Oslo, Norway
- Stavanger University Hospital, Stavanger, Norway
- Akerhus University Hospital, Lørenskog, Norway
- Gdynia Oncology Center, Poland,

- National Cancer Institute Warsaw, Warsaw, Poland
- Medical University of Silesia, Katowice, Poland
- University of Milan Bicocca, Milan, Italy
- Gemelli Hospital, Rome, Italy
- Bologna University Hospital Italy
- University Hospital Leuven, Leuven, Belgium
- Medical University of Graz, Graz, Austria
- Hospital Británico de Montevideo, Montevideo, Uruguay
- National Institute of Cancer Mexico

Sites working on approval include the University of Washington USA, Vancouver General Hospital Canada, University of Melbourne Australia, AC Camargo Cancer Center Brazil, and Karolinska University Hospital Stockholm Sweden. All participants will provide a written informed consent. The study protocol is registered in ClinicalTrials.gov (registration number NCT04294927).

References

1. Harmsen MG, Steenbeek MP, Hoogerbrugge N, van Doorn HC, Gaarenstroom KN, Vos MC, et al. A patient decision aid for risk-reducing surgery in premenopausal BRCA1/2 mutation carriers: Development process and pilot testing. Health Expect. 2018;21(3):659-67.
2. Steenbeek MP, van Bommel MHD, Harmsen MG, Hoogerbrugge N, van Doorn HC, Keurentjes JHM, et al. Evaluation of a patient decision aid for BRCA1/2 pathogenic variant carriers choosing an ovarian cancer prevention strategy. Gynecol Oncol. 2021;163(2):371-7.
3. Medeiros F, Muto MG, Lee Y, Elvin JA, Callahan MJ, Feltmate C, et al. The tubal fimbria is a preferred site for early adenocarcinoma in women with familial ovarian cancer syndrome. Am J Surg Pathol. 2006;30(2):230-6.
4. Casparie M, Tiebosch AT, Burger G, Blauwgeers H, van de Pol A, van Krieken JH, et al. Pathology databanking and biobanking in The Netherlands, a central role for PALGA, the nationwide histopathology and cytopathology data network and archive. Cell Oncol. 2007;29(1):19-24.
